# A Case of Bullous Rash Apparently Triggered by Meningococcal and Rotavirus Vaccines in an Infant: Focus on Infantile Bullous Pemphigoid

**DOI:** 10.3390/dermatopathology8010006

**Published:** 2021-02-23

**Authors:** Iria Neri, Valeria Evangelista, Alba Guglielmo, Andrea Sechi, Annalucia Virdi

**Affiliations:** Dermatology—IRCCS Policlinico di Sant’Orsola—Department of Experimental, Diagnostic and Specialty Medicine (DIMES), Alma Mater Studiorum-University of Bologna, 40138 Bologna, Italy; iria.neri@aosp.bo.it (I.N.); alba.guglielmo@gmail.com (A.G.); andrea.sechi@gmail.com (A.S.); annalucia.virdi@aosp.bo.it (A.V.)

**Keywords:** autoimmune bullous rash, bullous pemphigoid, infants, vaccine

## Abstract

Bullous pemphigoid (BP) is an autoimmune bullous disease and is a rare condition in childhood. Acquired tense acral bullae and fixed urticarial annular lesions on the trunk are diagnostic clues of infantile BP. Diagnosis is supported by immunosorbent assay (IgG anti-BP180 and BP230) and direct immunofluorescence (linear deposition of IgG at the dermo-epidermal junction). Topical and/or systemic corticosteroids are the first-line treatment. The prognosis is good with a self-limited clinical course. Differential diagnoses include impetigo and other bullous diseases in children, such as dermatitis herpetiformis, linear IgA bullous dermatosis and erythema multiforme. The etiopathogenesis is still unknown, and the role of antigen stimuli such as infections, drugs and vaccination is still debated.

## 1. Introduction

**Figure 1:**
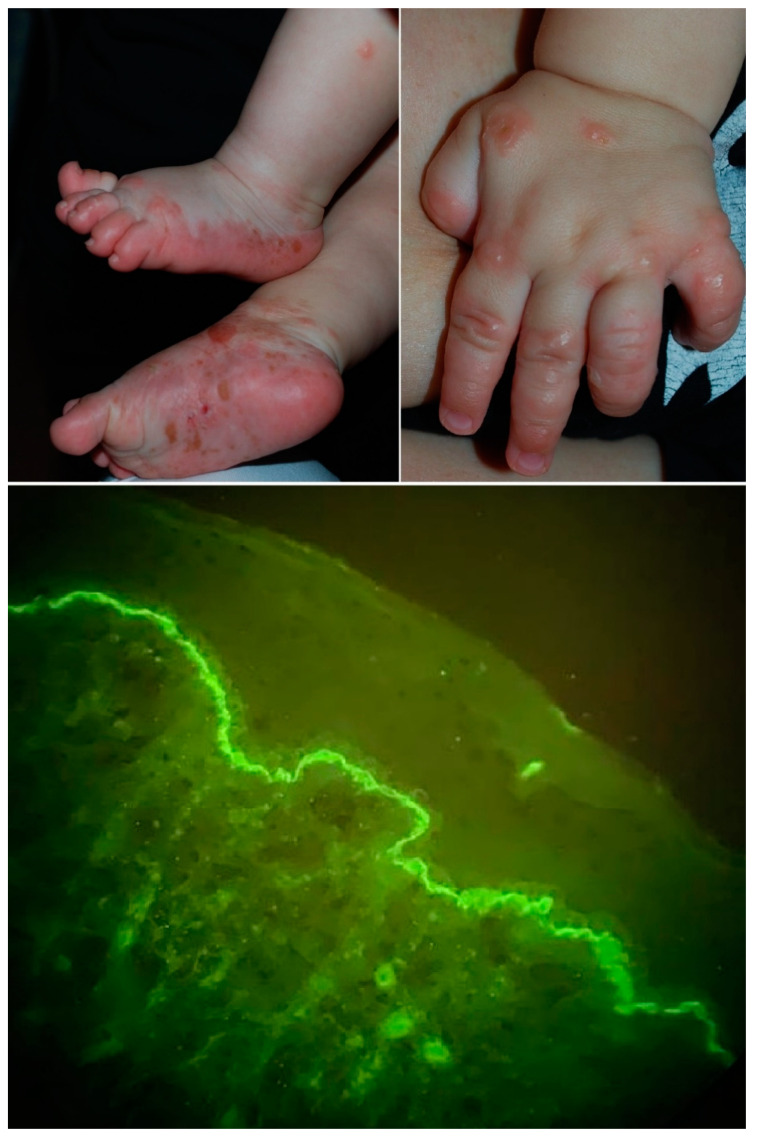
Bullae on the hands and feet. Direct immunofluorescence with linear deposition of immunoglobulin G (IgG) at the dermo-epidermal junction.

A four-month-old Caucasian boy was referred to us with a one-day history of bullous rash. He was otherwise healthy, and his family history was unremarkable. Physical examination revealed scattered, tense bullae on the feet, hands, limbs and trunk, and erythematous-edematous lesions on the face, without mucosal involvement (Figure 1). The lesions appeared three weeks after the meningococcal and rotavirus vaccines. Routine laboratory tests showed no abnormalities, and bacteriological and virological exams were negative. The immunosorbent assay (commercial ELISA, Biotest Laboratories Ltd., Underwood, Australia) showed positive values of IgG BP180 (205.20 U/mL, normal values < 20 U/mL) and BP230 (3.10 U/mL, normal values < 20 U/mL). Serological testing of the mother’s serum was negative. A 4 mm punch biopsy was made, and histopathology and direct immunofluorescence showed linear deposition of IgG at the dermo-epidermal junction (Figure 1), confirming the diagnosis of infantile bullous pemphigoid (BP). Direct immunofluorescence was negative for IgA deposition. Short-term treatment with oral corticosteroids (deflazacort 1 mg/kg/day) led to complete healing within two weeks. Six weeks after remission, our patient repeated the vaccinations without relapse.

## 2. Discussion

Bullous pemphigoid (BP) is a common acquired autoimmune subepidermal bullous disease that often affects elderly patients but is uncommon during childhood. BP is characterized by the production of autoantibodies against the basement membrane: immunoglobulin G (IgG) autoantibodies targeting BP 180 and BP 230 components of hemidesmosomes. Histopathology reveals a subepidermal blister with superficial dermal infiltrates of immune cells. Direct immunofluorescence (DIF) shows linear IgG and C3 deposits along the basement membrane [1,2].

The incidence of BP increases in patients older than 80 years. BP is considered a rare disease in children and even more infrequent in infants [2].

This bullous disorder is, in fact, fascinating due to its two different scenarios in pediatric age. Two variants with distinct peaks of incidence are reported. Infantile BP in the first year of life (53% of cases, with a median age of 4 months) and childhood BP at the age of 8 years (47% of cases, with median age 8 years). The former is characterized by palmoplantar and facial blisters and bullae, rarely generalized or associated with urticarial plaques on the trunk; the latter involves only the genital areas almost exclusively of females (vulvar BP) [3,4,5,6].

The etiology is still unknown. In particular, maternal antibodies and foreign antigens such as drugs, infections, and vaccines are speculated as possible triggers of infantile BP [5,6]. In the literature, a temporal correlation with vaccination was reported in infants under 1 year of age [6,7,8]. However, the latency between vaccination and clinical manifestations was variable, ranging from 5 h to 3 weeks; therefore, the triggering role is still debated [4]. According to some authors, the trauma created by the injection may cause blister formation through the inflammatory cascade activation mediated by the Th17/IL-17 pathway in genetically predisposed infants [8].

We can assume that injection into predisposed subjects may cause a transitory disruption of the basement membrane architecture with subsequent generation of anti-basement membrane-specific antibodies. This may explain the excellent clinical course of infantile BP.

Differential diagnoses include bullous impetigo and other blistering diseases such as dermatitis herpetiformis, linear IgA bullous dermatosis and erythema multiforme.

Oral corticosteroids are the first-line treatment and lead to healing from a few days to several months, and the prognosis is good. Some cases of mild recurrence were described after a new dose of vaccination; nevertheless, infant BP is a benign and self-limiting disease, so the vaccination must be continued [6].

Some authors have reported other treatments such as topical corticosteroids, dapsone, sulfapyridine, mycophenolate mofetil, and immunoglobulins [4].

## 3. Conclusions

In conclusion, infantile bullous pemphigoid represents a rare condition that must be investigated during the evaluation of acral bullous rash in infants. Infantile BP presents a favorable course compared to BP in elderly patients. The role of the vaccine as a risk factor is still debated, but vaccination is not contraindicated considering that infantile BP is a benign and self-limiting disease.
